# Into the Wild: Dissemination of Antibiotic Resistance Determinants via a Species Recovery Program

**DOI:** 10.1371/journal.pone.0063017

**Published:** 2013-05-22

**Authors:** Michelle L. Power, Samantha Emery, Michael R. Gillings

**Affiliations:** Department of Biological Sciences, Macquarie University, North Ryde, NSW, Australia; CSIRO, Australia

## Abstract

Management strategies associated with captive breeding of endangered species can establish opportunities for transfer of pathogens and genetic elements between human and animal microbiomes. The class 1 integron is a mobile genetic element associated with clinical antibiotic resistance in gram-negative bacteria. We examined the gut microbiota of endangered brush-tail rock wallabies *Petrogale penicillata* to determine if they carried class 1 integrons. No integrons were detected in 65 animals from five wild populations. In contrast, class 1 integrons were detected in 48% of fecal samples from captive wallabies. The integrons contained diverse cassette arrays that encoded resistance to streptomycin, spectinomycin, and trimethoprim. Evidence suggested that captive wallabies had acquired typical class 1 integrons on a number of independent occasions, and had done so in the absence of strong selection afforded by antibiotic therapy. Sufficient numbers of bacteria containing diverse class 1 integrons must have been present in the general environment occupied by the wallabies to account for this acquisition. The captive wallabies have now been released, in an attempt to bolster wild populations of the species. Consequently, they can potentially spread resistance integrons into wild wallabies and into new environments. This finding highlights the potential for genes and pathogens from human sources to be acquired during captive breeding and to be unwittingly spread to other populations.

## Introduction

Wildlife conservation strategies can present unrecognized threats. Captive breeding creates an atypical interface between humans, domestic animals and wildlife that potentially leads to exchange of microorganisms between these host groups. The translocation of animals between endangered populations removes barriers between previously isolated groups, allowing spread of pathogens that are novel for these groups, thus leading to emergence of new diseases. Consequently, actions taken during endangered species recovery programs can pose a significant risk for the transmission of disease. Despite this risk, translocation of wildlife regularly occurs with limited or no disease screening. Guidelines exist for minimizing disease transfer during translocation of wildlife [1], but only 24% of 700 translocations in Australia, New Zealand, Canada and USA incorporated a disease screening protocol [1].

Emergence of disease may be exacerbated by the spread of antibiotic resistant pathogens. There is evidence that proximity to human dominated ecosystems increases exposure to antibiotic resistance genes and the organisms that carry them [2], [3]. In contrast, resistance genes were not detected in enterobacteria isolated from wildlife in Finland or the Galapagos Islands [4], [5]. However, resistance determinants and resistant organisms can be found in areas far from the selection pressures imposed by antibiotics [6]. Transfer of resistant enterobacteria between chimpanzees and humans in Uganda has been reported [7], but no antibiotic resistance was detected in western lowland gorillas (*Gorilla gorilla gorilla*) experiencing increased exposure to humans [8]. Antibiotic resistance has been detected in wild and captive Iberian lynx *Lynx pardinus* [9] and Atlantic bottlenose dolphins *Tursiops truncatus* [10], as well as a wide range of wild fish, birds and mammals [6].

The rapid spread of antibiotic resistance has been facilitated by the mobility of DNA elements that carry genes encoding resistance [6]. One important means by which antibiotic resistance genes are acquired is via the activity of bacterial genetic elements called integrons [11]. Integrons encode an integrase (*intI*) that inserts gene cassettes at an integron-associated recombination site (*attI*), and drives gene expression with an adjacent promoter (P_c_). The most common integron in clinical pathogens is the class 1 integron. Class 1 integrons are also found in environmental bacteria, but those from clinical sources have unique structures, carry gene cassettes that usually encode antibiotic resistance, and are embedded in plasmids or transposons, a feature that facilitates their movement between cells and species [12]. Over the last 50 years, class 1 integrons have spread into many different bacterial species and have collectively acquired over 100 different resistance determinants [13], [14].

Exposure to human generated waste-water presents a pathway for transfer of bacteria and the antibiotic resistance genes they carry [15]. Captive or wild animals exposed to such sources can be colonized by microorganisms that are not typical of their natural habitats. Indeed, the majority of reports of class 1 integrons in vertebrates (gulls, flamingo, carp, salmon, and catfish) are typically associated with aquatic habitats [16], [17], [18], [19]. Environmental contamination with fecal material from domesticated animals and pets is also a risk factor [20]. Additionally, the use of antibiotics to treat or prevent disease in captive breeding facilities presents opportunities for selection of antibiotic resistant organisms [21].

Here we examined the penetration of integrons and their associated antibiotic resistance genes into the gut microbiota of a macropodid marsupial, the endangered brush-tailed rock-wallaby, *Petrogale penicillata.* This species was once widely distributed along the mountain ranges of Victoria, New South Wales and Queensland, Australia [22]. The brush-tailed rock-wallaby is listed as threatened in New South Wales under the NSW Threatened Species Conservation Act 1995 [23] and near threatened on the IUCN Red List of Threatened Species across eastern Australia [24]. In response to the status of the species, a National Recovery plan was instituted in 2005 [25]. The program is highly active and animals are often relocated between captive breeding facilities and wild populations [25].

## Materials and Methods

### Ethics statement

This research was undertaken in collaboration with the Office of Environment and Heritage and approved by Office of Environment and Heritage Animal Ethics Committee under permit numbers 050207/02 and 080728/01 and authorized by National Parks Scientific license No. 11934.

### Brush-tailed rock wallaby fecal sample sources

Brush-tailed wallabies inhabit steep rocky outcrops along the Great Dividing Range in South Eastern Australia. In response to a significant reduction in population size and range since European settlement [22] a number of conservation strategies have been instigated across Australia including captive breeding programs and translocations of both wild caught and captive bred individuals into wild populations [23] [26]. In NSW the main captive population (approximately 60 animals) is managed by Waterfall Springs Wildlife Sanctuary [23] which is located 70 km to the north of Sydney in Kulnara. Wild populations are found at a number of sites in national parks throughout NSW.

Brush-tailed rock-wallaby fecal pellets were collected by animal keepers or species recovery program personnel. Samples were collected during 2008-2009 from five wild populations in NSW Australia; Warrumbungles National park 31° 1′ 46.74′′ S, 148° 49′ 29. 60′′ E (Square Top mountain n  = 23 and Uringery n = 19), Oxley Wild Rivers National Park 30° 54′ 21.63′′ S, 152° 7′ 21. 37′′ E (Green Gully n = 7), Wollemi National Park 32° 47′ 29.58′′ S, 149° 41′ 26. 06′′ E (n = 12) and Capertee 33° 8′ 41.98′′ S, 149° 58′ 53. 21′′ E (n = 4). Each of the sampling sites supports a small wallaby population, ranging between 5 and 20 individuals per site. Samples were also collected from captive wallabies from a single breeding site at Waterfall Springs, NSW (n = 29). Fecal samples from wild animals were collected from the base of traps or feed stations within 8 hours of deposition. Pellets were placed into an airtight container and stored at 4°C for DNA extraction.

### DNA extraction and amplification of class 1 integron components

DNA was extracted from 75–150 mg of an individual fecal pellet using the Bioline Fecal PCR kit. Yield of DNA was assessed using agarose electrophoresis with Gel Red^TM^ post staining. The PCR competence of the resulting DNA was confirmed by amplification of 16S rRNA genes using primers f27 and r1492 (Table 1) [27].

**Table 1:** Primers used to amplify 16S rDNA and integron components.

Primer	Sequence 5′-3′	Target	Reference
f27	AGA GTT TGA TCM TGG CTC AG	16S rDNA	[27]
r1492	TAC GGY TAC CTT GTT ACG ACT T	16SrDNA	[27]
HS463a	CTG GAT TTC GAT CAC GGC ACG	*intI1*	[39]
HS464	ACA TGC GTG TAA ATC ATC GTC G	*intI1*	[39]
HS458	GTT TGA TGT TAT GGA GCA GCA ACG	*attI1*	[39]
HS459	GCA AAA AGG CAG CAA TTA TGA GCC	*qacEΔ*	[39]
GCU28	TCA GGC GTT ATT CAG TGC	*gcuF*	This study
OLFR155	CTG AAG GCT ACG CTG TCG AGT	*dfr*	This study

Initial screening for class 1 integrons was performed to detect the class 1 integron-integrase gene (*intI1*) using the primers HS463a and HS464 (Table 1). These primers target conserved regions within the integrase gene. Amplicons were resolved by electrophoresis on 2% agarose and samples generating a band of 473 bp were deemed *intI1* positive. Positive samples were then analysed using the primers HS458 and HS459 to identify gene cassette arrays. The cassette array is flanked by conserved regions (*attI1* and *qacEΔ1*) and HS458 and HS459 target these conserved elements (Table 1).

### DNA cloning and sequencing

Gene cassette PCR products were purified using Promega PCR purification columns and sequenced directly using the amplification primers HS458 and HS459. Where multiple bands were evident, indicating the presence of more than one class 1 integron, the amplified cassette arrays were ligated into T-tailed plasmid vectors (pCR2.1-TOPO; Invitrogen), and used to transform competent *E. coli* TOP10 cells. All procedures were as specified by the manufacturer (TOPO TA cloning kit; Invitrogen). Plasmids were purified using Qiagen plasmid purification kits and sequenced using the vector specific primers. For long cassette arrays (cloned from individuals WF15 and WF16), internal sequencing primers were designed. These were GCU28 for WF15 and OLFR155 for WF16. DNA sequencing reactions were performed at the Macquarie University sequencing facility using dye terminator technology. Sequences were determined on an Applied Biosystems 3130xl capillary sequencer, and were analyzed using bioinformatics software available through the Biomanager facility of ANGIS (http://www.angis.org.au/).

Sequences were annotated by hand after performing Blastn and Blastx searches through the NCBI website (http://www.ncbi.nlm.nih.gov/BLAST). Open reading frames within gene cassettes were identified using Blastx comparisons, and the boundaries of gene cassettes were identified using the core sequences (GTTRRRY) of the cassette recombination site *attC*. Sequences were prepared as GenBank flat files for database submission using Sequin v9.50. Sequences reported in this publication were lodged as Genbank accession numbers GU060314-GU060323.

## Results

### PCR screening

DNA was successfully extracted from 94 brush-tailed rock-wallaby fecal samples (wild  = 65 and captive  = 29). PCR using 16S rDNA primers f27 and r1492 resulted in bands of the expected size, establishing that all samples were PCR competent. The presence of the class 1 integron-integrase gene, *intI1*, in fecal samples was determined by PCR screening with primers HS464/HS463a. The *intI1* gene was not detected in any wild brush-tailed rock-wallaby sample (n = 65) representing five independent populations. In contrast, 48% of samples from captive rock wallabies (14/29) generated strong, single bands consistent with the expected size of 473 bp for *intI1*. Sequencing of these PCR products confirmed their identity as *intI1* and that the *intI1* sequence was identical to those found in class 1 integrons from human clinical isolates. Samples positive for *intI1* were then screened using the primers HS458/HS459 to amplify the associated gene cassette array. These primers targeted conserved regions that flank the gene cassettes, and generated amplicons that varied in size depending on the gene cassette content of the class 1 integron.

### Cassette arrays

**Figure 1:**
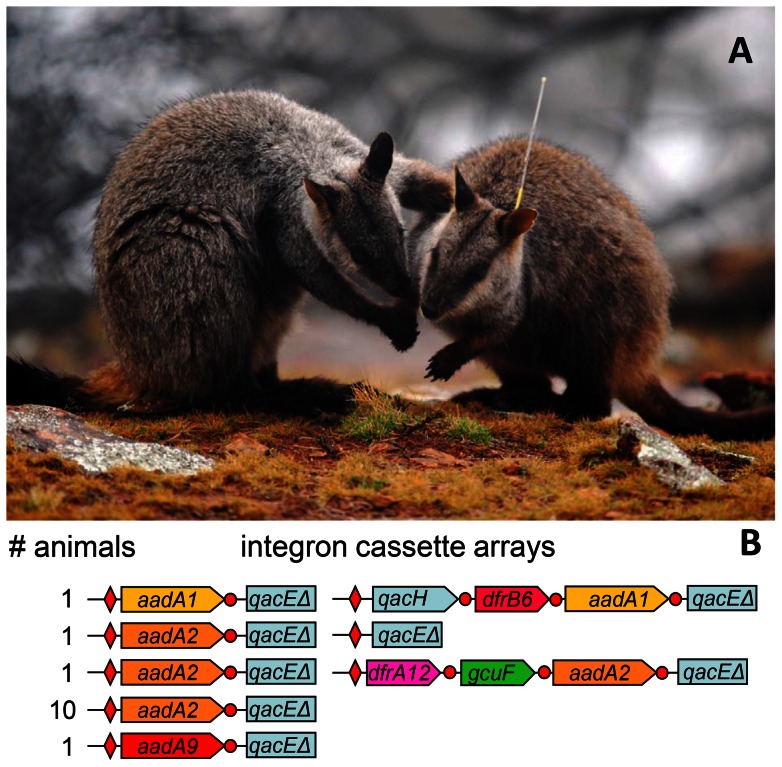
Antibiotic resistance integrons in feces from captive rock wallabies. (**A**) A wild brush-tailed rock-wallaby meets an animal released from a captive breeding program (on right, with radio tracking collar). Photo Credit: Hugh McGregor (**B**) Schematic maps of integron cassette arrays recovered from 14 of 29 captive wallabies. Numbers of wallabies with each array combination are shown. Red diamonds – the primary integron recombination site, *attI1*; red circles – gene cassette recombination sites, *attC*; broad arrows – genes showing direction of transcription. Gene symbols are as follows: *aadA* genes encode aminoglycoside adenyltransferases that confer resistance to streptomycin & spectinomycin; *qac* confers resistance to quaternary ammonium compounds, *dfr* genes encode dihydrofolate reductases that confer resistance to trimethoprim, *gcuF* unknown function (*5*).

Six different integron gene cassette arrays were detected in the 14 captive rock wallabies (Figure 1, Table 2). The most common cassette array contained the single *aadA2* gene cassette, and was identified in 12 animals. Additional integrons, isolated from individual wallabies, contained the single gene cassettes *aadA1* and *aadA9* (Table 2). Three animals contained at least two distinct cassette arrays (Figure 1).

**Table 2:** Class 1 integrons identified in captive rock wallabies from Waterfall Springs.

Animal Microchip	*intI1* ^A^	Cassette array(s)^B^	Accession #
982009105312751	Positive	*aadA2*	
982009105311668	Positive	*aadA2*	GU060315
982009104973684	Positive^C^	*No cassette*	GU060317
		*aadA2*	GU060316
982009104994605	Positive^C^	*aadA1*	GU060318
		*qacH dfrB6 aadA1*	GU060319
982009105012855	Positive^C^	*aadA2*	GU060320
		*dfrA12 gcuF aadA2*	GU060321
982009101489794	Positive	*aadA2*	
982009101346340	Positive	*aadA2*	GU060322
982009104985227	Positive	*aadA2*	
982009101168950	Positive	*aadA9*	GU060323
982009104984291	Positive	*aadA2*	
982009101270507	Positive	*aadA2*	
982009104848629	Positive	*aadA2*	
982009104894764	Positive	*aadA2*	GU060314
982009104800191	Positive	*aadA2*	

Presence of the class 1 integron-integrase gene, *intI1*, as determined by PCR with primers HS464/HS463a (A) and the identity of integron gene cassettes as determined by sequence analysis of cassette arrays amplified with primers HS458/HS459 (B). Two distinct cassette arrays were detected in some wallaby samples (C).

## Discussion

Here we identified the penetration of class 1 integrons and their associated antibiotic resistance genes into the gut microbiota of the endangered brush-tailed rock-wallaby using PCR screening. There have been few studies examining antimicrobial resistance in Australian wildlife. Low levels of antibiotic resistance have been detected in Enterobacteriaceae from various marsupial species using culture based susceptibility methods [28]. However, the bacterial strains from Australian marsupials were more susceptible to antibiotics than strains isolated from marsupials in Mexico [29].

Culture-based methods and antimicrobial susceptibility testing are commonly used to identify antibiotic resistance in wildlife species [5], [8], [9], [10]. Culture-based approaches have screening biases [30]. For example the most abundant strains in a fecal microbial community will be selected during enrichment, potentially outcompeting growth of the rare organisms or strains that may represent microbial species or strains with mechanisms that confer resistance. Additionally, sampling of wild animals is often difficult, and non-invasive sampling via opportunistic collection of feces is commonly used. In these cases sample age cannot always be guaranteed and hence bacterial culture may not be optimal. Screening for the molecular signatures of antibiotic resistance as performed in this study is potentially a more sensitive approach to detecting antimicrobial resistance in the microbiota of wildlife.

Using molecular testing, six different gene cassette arrays were detected in fecal material from captive rock wallabies. The associated integron-integrase gene sequences detected in the rock-wallaby microbiome were identical to those from clinical class 1 integrons associated with human pathogens and commensals [13]. The cassette arrays detected are amongst those most commonly recovered from human pathogens [13]. Three of the six cassette arrays in rock wallabies have also been identified in beef cattle reared in Australia [31], [32]. The presence of sequence signatures typical of class 1 integrons from clinical bacteria and domestic animal sources strongly suggests that the wallabies acquired their integrons from sources contaminated with human or domestic animal fecal bacteria. Class 1 integrons are disseminated via human waste streams into wastewater treatment plants, and then into aquatic environments such as rivers and estuaries [33], [34], [35]. There are numerous reports of class 1 integrons in animals associated with these aquatic environments [17], [18], [19].

How the class 1 integrons made their way into the wallaby microbiota is unknown, but it seems likely that water or feed may have acted as a conduit for bacteria carrying these integrons. Drinking water provided to wallabies is pumped from underground springs. Food sources include Lucerne chaff and a commercially prepared macropod pellets which are supplemented with fresh vegetables. The wallabies are housed in outdoor enclosures that have varying degrees of run off after periods of high precipitation. Groundwater contamination, contamination during processing or vegetable production, or contaminated run-off all provide possibilities for exposure to microorganisms from human or animal sources.

The diversity of cassette arrays detected within the class 1 integrons indicates that captive wallabies acquired integrons or new gene cassettes on a number of independent occasions. To account for this acquisition sufficient numbers of bacteria carrying diverse integrons would need to be present in the general environment occupied by the wallabies. Diverse cassette arrays have been detected in aquatic environments and animals associated with aquatic habitats [18]. The wallabies in this study were bred and reared in captivity for a period of up to four years, allowing prolonged exposure to sources of class integrons, and sufficient opportunity for bacteria from these sources to colonize captive animals.

The identification of class 1 integrons and associated antibiotic resistance mechanisms in fecal DNA from captive animals has implications for successful animal management. There is a potential risk for future disease control and animal treatment regimes, in that cassette arrays contained genes that encode resistance to spectinomycin, streptomycin and trimethoprim. These antibiotics are commonly used in veterinary practice, and listed by the World Organisation for Animal Health as antimicrobials of veterinary importance (http://www.oie.int/). The loss of ability to successfully treat disease is detrimental to endangered species recovery programs. It should be noted that the captive wallabies described here acquired their integrons in the absence of antibiotic therapy.

Further, integrons are mobile DNA elements that can be exchanged within host associated microbes and move between host microbes and pathogens. Exchange of microbes between different hosts can also transfer these elements to new hosts [6]. The captive wallabies identified as carrying class 1 integrons in their gut microbiota have now been released into a wild rock-wallaby population. Consequently, translocated wallabies carry class 1 integrons with them and may potentially spread integrons into wild individuals and into new environments via fecal deposition. This finding highlights the potential for pathogens to be acquired during captive breeding and to be unwittingly spread to other populations and other species. The transmission of microbiota carrying class 1 integrons to from mother to offspring in humans [36] highlights potential for further spread of class 1 integrons through captive breeding practices. Class 1 integrons maybe potentially passed from mother to offspring or through animal relocation to other captive facilities, a practice used to ensure genetic diversity in endangered species [37]. The detection of class 1 integrons in captive rock wallabies further highlights the risk of disease in conservation practice [38].

Disease management is central to species recovery programs, but is often secondary to habitat restoration and animal breeding. The detection of class 1 integrons from anthropogenic sources demonstrates the risks of disease transmission at the wildlife – human interface. The release of wallabies and the class 1 integrons into wild habitat provides the opportunity to monitor dissemination of class 1 integrons and their associated genes cassettes through individual wallabies, the population and the environment, providing a unique opportunity to study the fate of resistance genes in a natural setting.

Our study has confirmed the potential for transmission of disease organisms at the human, domestic animal and wildlife interface [1]. To reduce the threat of disease through conservation practice, routine pathogen screening must be considered as an essential component of a management plan. Without such screening, animal translocation may alter host-pathogen interactions, further threatening already endangered species.

## Acknowledgments

We would like to thank Celia Thomson from Waterfall Springs and Deborah Ashworth and Todd Soderquist from the Department of Environment, Climate Change and Water for provision of samples. We would also like to thank Nichola Hill for providing valuable feedback in manuscript. Photo Credit figure 1: Hugh McGregor.
